# Accessing care for Long Covid from the perspectives of patients and healthcare practitioners: A qualitative study

**DOI:** 10.1111/hex.14008

**Published:** 2024-03-14

**Authors:** Fidan Turk, Jennifer Sweetman, Carolyn A. Chew‐Graham, Mark Gabbay, Jessie Shepherd, Christina van der Feltz‐Cornelis, STIMULATE‐ICP Consortium

**Affiliations:** ^1^ Department of Health Sciences University of York York UK; ^2^ School of Medicine Keele University Keele UK; ^3^ Department of Primary Care and Mental Health University of Liverpool Liverpool UK; ^4^ NIHR ARC NWC Liverpool UK; ^5^ Department of Education University of York York UK; ^6^ Hull York Medical School (HYMS) University of York York UK; ^7^ Institute of Health Informatics University College London London UK

**Keywords:** access to care, lived experiences, Long Covid, long‐term conditions, multiple symptoms, qualitative study, waiting times

## Abstract

**Background:**

Long Covid is an emerging long‐term condition, with those affected raising concerns about lack of healthcare support.

**Objective:**

We conducted a qualitative study to identify facilitators and barriers to healthcare access for people with Long Covid, aiming to enhance our understanding of the specific nature of these barriers and how patient experiences may vary.

**Setting and Participants:**

In the context of the Symptoms, Trajectory, Inequalities and Management: Understanding Long‐COVID to Address and Transform Existing Integrated Care Pathways (STIMULATE‐ICP) Delphi study, a nationally distributed online survey was conducted. Eight patients and eight healthcare practitioners (HCP) were interviewed via telephone or video call. Framework analysis, sensitised by the candidacy theory, was used to identify barriers and facilitators over four levels of access to care.

**Results:**

Three themes were identified: (i) patients' efforts to navigate emerging pathways for Long Covid, (ii) the patient–HCP interaction and (iii) service resources and structural constraints. Barriers to specialist care included long waiting times, communication gaps across services and a lack of continuity in care. Facilitators included collaborative, patient‐centred approaches, patients' active role in their healthcare and blended approaches for appointments. The perspectives of both patients and HCPs largely aligned.

**Discussion:**

The candidacy framework was valuable in understanding the experiences of people with Long Covid seeking access to healthcare. Individuals perceived themselves as eligible for care, but they often encountered obstacles in obtaining the expected level of care or, in some cases, did not receive it at all. Our findings are discussed in the context of the candidacy model through multiple processes of identification, negotiation, permeability and appearances at health services. These themes seem to be especially important for the emerging new pathway model and are relevant to both primary and secondary care.

**Conclusions:**

This study highlights that despite these interviews being conducted two years after the start of the COVID‐19 pandemic, people with Long Covid still struggle to access healthcare, emphasising the ongoing need to provide equitable timely healthcare access for people with Long Covid.

**Patient or Public Contribution:**

People with Long Covid advised on all stages of this research.

## 1 INTRODUCTION

Long Covid, a newly emerging long‐term condition, has significantly impacted a considerable number of people, as reported by the Office for National Statistics.^1^, ^2^ A recent study suggests that between 325 and 606 million people^3^ would probably live with long COVID around the world. By March 2023, approximately 1.9 million individuals reported experiencing symptoms persisting for at least 4 weeks after an acute COVID‐19 infection in the United Kingdom (UK).^2^ Common symptoms include fatigue, brain fog, paraesthesia, chest pain and palpitations, muscle and joint pain and shortness of breath.^2^, ^4^ The National Institute for Health and Care Excellence (NICE) guideline defines persisting symptoms as both ongoing symptomatic COVID‐19 (lasting from 4 to 12 weeks) and post‐COVID‐19 syndrome (lasting for more than 12 weeks and not explained by an alternative diagnosis).^5^ Long Covid is the patient‐preferred term due to the ongoing nature of symptoms^6^; this term is used throughout this paper. It has been suggested, both in the UK and globally,^7^, ^8^ that Long Covid is likely to pose a significant burden on the health service^9^ and the UK economy, with an increased likelihood of long‐term absence from work or economic inactivity among those affected.^10^ The investment in Long Covid services should provide access to specialist care,^8^ but current availability and accessibility of these services varies.

Recent reports emphasise demand exceeding capacity.^11^, ^12^ In the case of specialist care, long waiting times and strained services have been reported.^13^ One recent study reported that individuals with Long Covid have struggled to access sufficient healthcare support^14^; another study suggested that one‐third of people with Long Covid^15^ who have been referred are still awaiting appointments with Long Covid services.

Accessing healthcare services has been reported to present challenges in the pre‐ and post‐COVID context, including for Long Covid.^16^ Given the increased demand for healthcare^9^ and the emerging nature of Long Covid as a long‐term health condition, the identification of barriers and facilitators to healthcare access is essential.^15^

**Figure 1:**
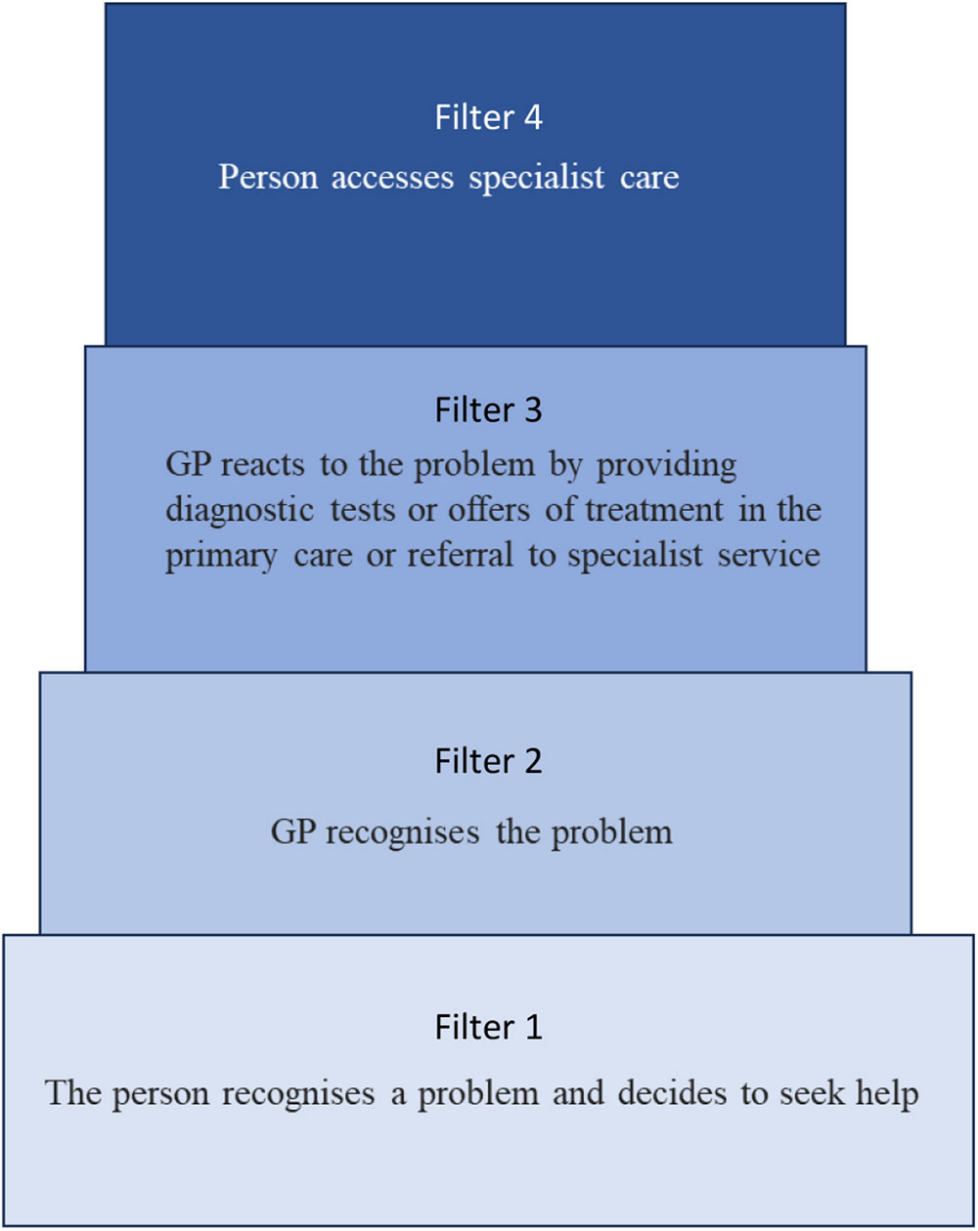
Pathways to Care Model for Long Covid access. Model based on previous research.^15^ GP, General Practitioner.

**Figure 2:**
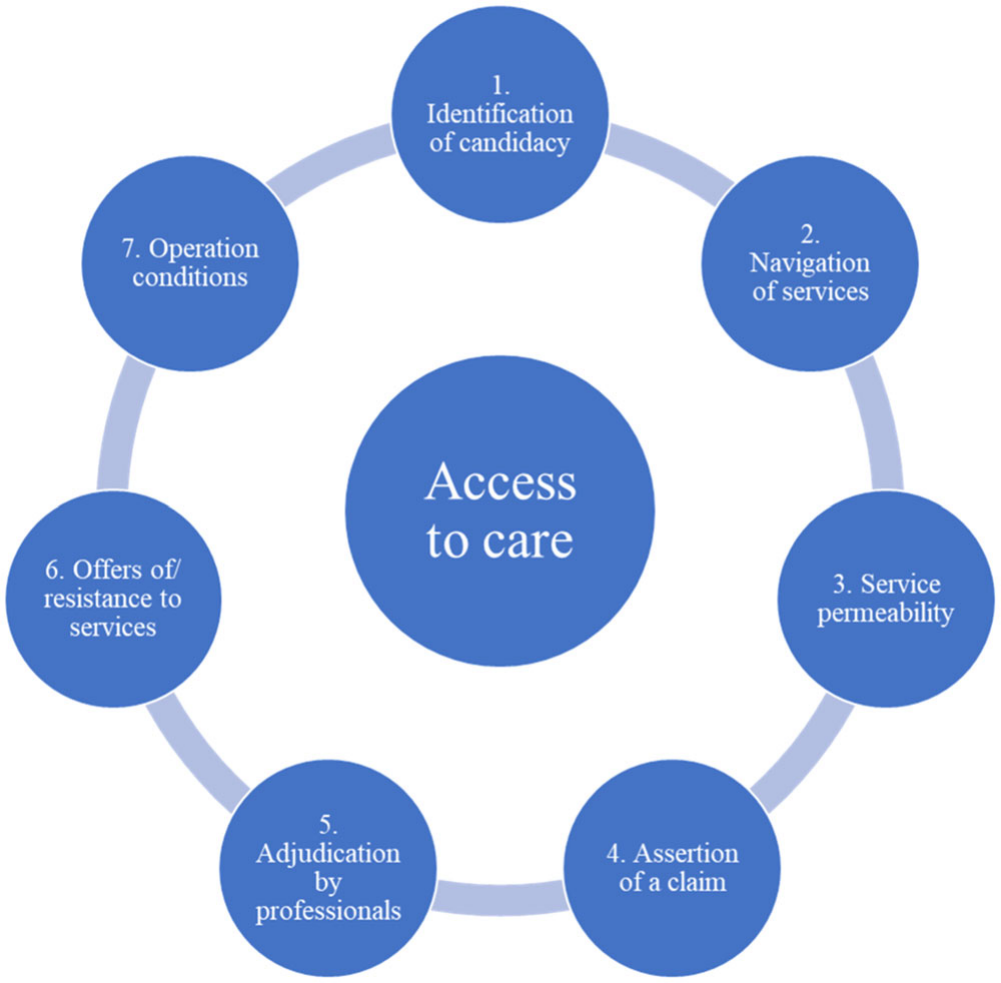
The stages of the Candidacy Framework.

‘Access to health care’ is a complex multifaceted process that consists of one's path to care seeking, the point of entry into the healthcare systems, and use of services within that system.^17^ The pathways‐to‐care model that was adapted for Long Covid in the UK suggests that individuals who seek healthcare pass through four filters in the healthcare system.^15^, ^18^ These filters reflect: (1) the person recognises a problem and decides to seek help (2) General Practitioner (GP) recognises the problem (3) GP reacts to the problem, providing diagnostic tests, treatment in primary care or referral to specialist service (4) person accesses specialist care (Figure 1). Building on the pathways‐to‐care model, the candidacy framework suggests that access to healthcare is often portrayed as a process that requires effort from patients to attain, and eligibility for accessing care is an ongoing negotiation within patient–practitioner interactions.^17^ The candidacy framework is used to understand how people assess their eligibility for accessing health services and how they legitimise their interaction and engagement with services. Its aim is to offer a deeper insight into the factors influencing individuals' perceptions of eligibility. This framework includes seven stages of an individual's journey to access (Figure 2). This concept has been extended to a range of health conditions such as mental health problems, multiple sclerosis and antenatal care^19^, ^20^, ^21^; however, it has not yet been extended to Long Covid. This study aims to address this gap by utilising these models to inform data generation and analysis.

This paper aims to apply the pathways to care model and the candidacy framework to thoroughly examine the barriers and facilitators in accessing care for individuals with Long Covid. This paper reports the findings of a semi‐structured interview study exploring the perspectives of patients and healthcare practitioners (HCPs), offering an understanding of factors that influence the journey of care‐seeking and the determination of candidacy within the context of Long Covid. By exploring the challenges and facilitators faced when attempting to access healthcare, the findings will inform policy changes to formulate targeted and effective approaches to improve care access and delivery.

## 2 MATERIALS AND METHODS

### 2.1 Ethics

This work was part of the Symptoms, Trajectory, Inequalities and Management: Understanding Long Covid to Address and Transform Existing Integrated Care Pathways (STIMULATE‐ICP) Delphi study, which was reviewed and approved by the University of York Health Sciences Research Governance Committee on 17 December 2021 (HSRGC/2021/478/A: STIMULATE).^22^

### 2.2 Design

This was a sub‐study of the STIMULATE‐ICP‐DELPHI. Details of the STIMULATE‐ICP studies can be found on the study website.^23^ The Delphi study protocol was published elsewhere.^24^ For this component, we utilised semi‐structured interviews to examine the experience, and needs for treatment, of people living with Long Covid. We undertook this study following the COnsolidated criteria for REporting Qualitative research (CORE‐Q) and the standards for reporting qualitative research; the CORE‐Q checklist is in the Supporting Information Material.^25^, ^26^

### 2.3 Recruitment

Recruitment for the interviews was nested in the STIMULATE‐ICP‐DELPHI recruitment. From a group of survey participants who had expressed a willingness to participate in an interview, participants were selected using a maximum variation approach. Using details from the expression of interest form, the research team chose a purposive sample, consisting of people living with Long Covid and practitioners. The minimum target for this combined sample size was set at 10–15 participants to ensure a diverse representation of experiences. People living with Long Covid were selected to provide a wide variety of symptom experiences to reflect a broad range of patient experience. HCPs were selected from those who expressed an interest across a wide range of specialties, incorporating experiences from primary and secondary care. Recruitment ceased once authors deemed that the qualitative data collected provided sufficient ‘information power’ to address the research questions.^27^ We selected people with different symptoms and from different locations to increase breadth of sample, reflecting the variety of health services and the experience of different symptoms (purposive sampling).^28^

### 2.4 Participants

Eligibility criteria for patients were: (a) adults aged 18 and above, (b) individuals with lived experience of Long Covid, (c) residents of the UK at the time of data collection, and (d) the capacity to provide informed consent.

For HCPs, the criteria were: (a) adults aged 18 or over, (b) currently offering care to people with Long Covid in the UK and (c) the capacity to provide informed consent. Efforts were made to reflect different healthcare settings, that is, GPs for primary care, and specialists for specialist care for long‐term conditions, as well as Long COVID clinics, to enable the exploration of barriers and facilitators of the patient trajectory through care.

Participants were deemed ineligible if they were: (a) individuals aged 17 or younger, (b) family members, caregivers or friends of individuals with Long Covid or a long‐term condition, (c) residing outside of the UK or (d) unable to provide consent for research.

In total, 47 people expressed an interest in participating. Participants were selected to maximise sample variation based on (i) patient symptoms or HCP specialty, (ii) geographical location, (iii) demographic characteristics. Twenty‐six patients and eight HCPs were deemed eligible; patient participants not initially selected for interview were retained for the duration of the interviewing period in case of participant withdrawal or to accommodate further data collection. Two patient participants were selected for inclusion but were unable to participate in the interview due to illness. After interviewing eight patients and eight HCPs, no new codes were identified during the ongoing process of our framework analysis indicating that data saturation was achieved.^29^ Table 1 shows the demographic characteristics of participants, and Table 2 shows the characteristics of HCPs. The mean (*M*) age was 49.83 years (standard deviation [SD] = 11.1) for HCPs and 39.63 years (SD = 8.98) for patients. Most participants were White and from England. Selection for participation was stopped once saturation was achieved.

**Table 1:** People living with Long Covid demographics.

Participant number	Gender	Age range	Ethnicity	The type of care patients seek help, and whether issues with accessing care are still ongoing or resolved	
Patient 1	Male	40–49	White	Access to GP–No assistance.	
				A&E.	
				Private testing.	
Patient 2	Male	50–59	White	Access to GP–Blood test with no further action by the GP.	
				Waited for referrals for 14 months.	
Patient 3	Female	30–39	Other ethnic group	During the initial three GP appointments:	
				First appointment, treatment didn't help, waited 3 weeks.	
				Second appointment, no relief, another 3‐week wait.	
				Pushed for referrals and got referred to a LC clinic.	
				After waiting 12 weeks, discovered a mistake with the documents.	
				Switched to a new GP, referred to neurorehabilitation service and IAPT, but developed new symptoms. Another GP referred to cardiology in just 4 weeks, then to neurology.	
				Discharged from one LC clinic and re‐referred to another LC clinic.	
Patient 4	Female	30–39	Asian or Asian British	GP visits initially unproductive.	
				Referred to LC clinic by 111. However, no symptoms appointment with clinic.	
				Subsequent visits to GP and a private doctor.	
				Finally, a helpful GP referred to the rapid diagnostic team.	
Patient 5	Male	40–49	White	Referred to LC clinic by GP, but it wasn't helpful.	
				Needed a neurology referral but couldn't get it through the LC clinic.	
				After a long wait, finally saw a neurologist in a different city, but the appointment was in an MS clinic.	
				Currently waiting for a referral to the neurology clinic	
Patient 6	Male	30–39	White	Two GP appointments, but no referral to the LC clinic.	
				Sought private care.	
				Switched to a different GP who referred to the LC clinic, although the help received didn't fully meet his needs.	
				Also received a referral to mental health services.	
Patient 7	Male	40–49	White	First GP visit provided no help.	
				Another GP, after 5 months, recognised the issue and referred to the LC clinic. No response from the clinic.	
				Moved to another city, visited a new GP, and obtained another referral to a LC clinic.	
				Waited for 6 months and had 15‐min phone appointments.	
				Eventually received a neurology referral from GP, which included website links.	
				The GP remained helpful throughout.	
Patient 8	Female	20–29	White	Visited GP and A&E, advised to see GP, and underwent some tests.	
				Referred to LC clinic but still waiting for an appointment.	

*Note*: All were from England apart from one.

Abbreviations: A&E, accident and emergency; GP, General Practitioner; IAPT, improving access to psychological therapy; LC, Long COVID; MS, multiple sclerosis.

**Table 2:** Healthcare practitioners' demographics.

Participant number	Gender	Age range	Ethnicity	Healthcare service	Condition that HCPs provide care for
HCP 1	Female	20–29	Mixed or multiple ethnic groups	Work in the post‐COVID service and doing clinical assessment of patients referred to	People with LC
HCP 2	Male	50–59	Black, Black British, Caribbean or African	Consultant in Rehabilitation Medicine Secondary/specialty care	LTCs including people with LC
HCP 3	Female	50–59	White	GP	People with LC
				Community care	
HCP 4	Female	50–59	White	GP and clinical lead for post‐Covid clinic	People with LC
				Primary care	
HCP 5	Female	Missing data	Missing data	Missing data	People with LC in post‐covid clinic
HCP 6	Female	50–59	White	Clinical lead and Manager	LTCs including people with LC
				Community care	
				Specialist	
HCP 7	Male	60–69	White	GP	People with LC
				Primary care	
HCP 8	Male	Missing data	Missing data	Secondary/specialty care	People with LC

*Note*: All from England apart from two missing data.

Abbreviations: GP, General Practitioner; HCP, healthcare practitioner; LC, Long COVID; LTC, long‐term condition.

### 2.5 Procedure

All participants received information about the study and gave informed consent. Interviews were conducted (J.Sw. and J.Sh.) between May and July 2022 by telephone or Zoom^R^. Topic guides were used to guide conversation (see Table 3). The topic guide was developed by the research team with guidance from the moderator panel, which included two patient and public involvement (PPI) members and was part of the STIMULATE‐ICP Delphi study.^24^ Interviewers were introduced as researchers working on the project who were interested in finding out about experiences of care for people with Long Covid and of those offering care across multiple settings. Participants were offered flexibility for their interview to take place over two time‐periods, or to shorten the length of the interview if needed for any reason; however, no participants requested this. During data collection, internet signals were poor in some instances and associated interviews changed to telephone from online interviews part‐way through; no other changes to agreed plans were made. No participants were interviewed twice. No field notes were made during or after the interviews.

**Table 3:** Interview topic guide.

Group	Patients	HCPs
Questions	How long have you had Long COVID?	Can you please describe the service you offer to people with Long COVID/long‐term conditions?
	If relevant, what was the timescale between you seeking support and receiving care?	What impact do you think this has for patients?
	What do you think about the time it took to get into the service?	What works well?
	Please tell me about any contacts you've had with health or other care services as a result of your Long COVID.	What could be changed?
	What do you think that service did well?	What would you need to improve your services?
	What do you think could be improved?	

Abbreviation: HCP, healthcare practitioner.

Interviews lasted between 36 min and 1 h (for HCPs: *M* = 47 min, SD = 8.2; for patients: *M* = 47 min, SD = 5.9). Audio recordings were transcribed verbatim into Microsoft Word documents (F.T., N.S., J.Sh. and J.Sw.). Transcripts were not returned to participants for comment and/or correction. Identifiable information was removed from transcripts to prepare for analysis. All data were electronically stored on the University of York secure server, with access restricted to the research team and requiring a password for data access.

### 2.6 Analysis

Anonymised and corrected transcripts were stored, and analysis was supported by NVivo software (Version 12).^30^ A Framework approach^31^ was used for analysis. The use of framework analysis, considered appropriate for investigating our research questions, facilitates the review and refinement of ideas. A framework approach allows for a structured and documented analysis procedure that can be made accessible to multiple researchers within a team.^32^ The analysis process was iterative, developing through discussions within our multidisciplinary team.^32^ Framework analysis is a qualitative method uniquely suited for applied research because it allows researchers to classify the codes into facilitators or barriers, comparing patient and HCP perspectives.^33^

After familiarisation with the transcripts, initial codes were applied to transcripts (F.T.). To enhance the trustworthiness of the analysis, J. Sw. independently coded 10% of transcripts, with any disagreement in coding resolved through discussion. The research team (C.F.C., F.T. & J.Sw.) met regularly to discuss and engage with the codes and transcripts. Themes developed through discussion and examination of the coded transcripts.

### 2.7 Patient or public contribution

The STIMULATE‐ICP parent study has been enriched by robust PPI using multiple channels, including regular updates and webinars, surveys and social media. The STIMULATE‐ICP DELPHI sub‐study, the setting for this qualitative study, has been informed by existing engagements with people with experience of Long Covid. PPI co‐applicants and the larger PPI group contributed to conceptualising the research questions, topic guide, recruitment, analysis plan and the manuscript describing the results.

In addition, people with relevant disease experience were involved in the expert panel of the Delphi study. Members of the public and patients were involved as stakeholders for this project, increasing awareness with relevant groups, promoting research activities, and drafting recommendations in relation to this work. PPI co‐applicants and the larger PPI group advised on all aspects of the study.

### 2.8 Reflexivity

Both interviewers (J.Sw and J.Sh) are female academic researchers working on this STIMULATE‐ICP sub‐study. J.Sw. holds a PhD and works as a research associate, J. Sh. was a PhD candidate and worked as a research assistant. C.F.C. is a professor of psychiatry and holds MD and PhD degrees. F.T. holds a PhD and worked as a research associate. All members of the analysis team are females. The analysis was conducted by researchers with experience conducting qualitative research, and research in topics such as health psychology, psychiatry, medicine and mental health.

Except for two HCP participants, researchers had no prior relationship with any participants at the time of study commencement. Two HCPs interviewed for this study were members of the wider STIMULATE‐ICP consortium but had no input into the design or conduct of this qualitative study. None of the research team members have been diagnosed with Long Covid.

## 3 RESULTS

Three themes influenced participants' experiences with access to healthcare: (1) patients' efforts to navigate emerging care pathways for Long Covid (2) interactions between patients and HCPs and (3) service resources and structural constraints. Table 4 summarises facilitators and barriers to access to care categorised by themes.

**Table 4:** Facilitators and barriers to access to care categorised by themes.

	Facilitators	Barriers
Theme 1: Patients' efforts to navigate emerging pathways for Long Covid	Engagement with online communities	Unmanaged online resources
	Pushing for tests, treatment, and referral from GP and specialist	GP being overwhelmed
	Taking an active role in healthcare management	
	Pushing for tests, and treatment from specialist	
Theme 2: The interaction between patients and HCPs	A collaborative and patient‐centred approach	Limited knowledge about Long Covid
	The continuity of care and trust between patients and HCP	Doctors' unresponsive approach
	GPs having prior knowledge of the patients	
	GPs' open‐mindedness and willingness	
Theme 3: Service resources and structural constraints	Electronic booking and reminder systems	Oversubscribed GPs
	Alternative ways of contact	Lack of variety in appointments (remote, in person)
	Signposting self‐management services during waiting for referrals time	Lack of continuity in care
		Long waiting times for specialist
	A single point of access for triaging the referrals	Lack of communication across various services

Abbreviations: GP, General Practitioner; HCP, healthcare practitioner.

### 3.1 Patients' efforts to navigate emerging pathways for Long Covid

Many participants described efforts to navigate, and difficulties in their encounters with, care pathways for Long Covid. Three patients described their symptoms as beginning during the early stages of the COVID‐19 pandemic, when there was limited knowledge about how the acute infection could cause ongoing and lasting symptoms (which came to be called Long Covid) and no pathways existed for those with ongoing symptoms. Consequently, many patients reported having to negotiate their own care pathways.

#### 3.1.1 Online resources and support groups

Patients found support and advocacy through online support groups, using these platforms to access resources about their condition.

No formal advice on it at all. It's mostly been me [online group] that's helped and like resources around that about pacing and how to actually do that. (P‐4)

Patients reported the value of connecting with others who have Long Covid for emotional support, a better understanding of their condition, and to share information about navigating the healthcare system.

Because I think it's hard to try and understand this condition unless you've got it or unless you work in healthcare and you see … in healthcare and you see it every day so … yeah being able to connect with other people that have Long Covid and just talk out, you know vent and rant and you know talk out what we feel, that has been really great and yeah it would have been nice to connect with people earlier. (P‐3)

The importance of peer support for people with Long Covid was highlighted by HCPs.

I think them hearing other people and talking to other people who have had similar experiences so being able to hear other people's stories will be helpful, so that peer support. (HCP‐6, specialist)

Despite the increased availability of online information, HCPs recognised that such information may not be reliable, or evidence based. HCPs suggested that this would contribute to a sense of confusion among those seeking information or help.

Compounded by the confusion which is generated by people engaging with dubious healthcare sources online and so much time is spent sort of navigating erroneous opinions, bizarre theories with patients… (HCP‐8, secondary care)

None of the patient participants mentioned negative effects of the online communities, although some noted that they could not engage in online communities due to their symptoms, particularly brain fog and fatigue.

#### 3.1.2 Primary care

Patients described the challenges of being believed and having their symptoms recognised by GPs. For some people with Long Covid, finding a GP knowledgeable about Long Covid helped them gain a diagnosis.

It was probably about 11 months before somebody actually recognised, it was long‐Covid, which was a locum GP who is standing in for my regular GP at the time. And who is far more knowledgeable about long‐Covid, she'd been to a few conferences about it and just immediately said ‘yep, that's long‐Covid’. (P‐7)

While most people with Long Covid in our study described being able to book an initial GP consultation, some patients felt they had to actively advocate for further investigations, assessments, treatment, or referrals.

I had to really push and say, can you please refer me to a Long Covid clinic. (P‐4)

Some patients reported that they had to take on a very active role in advocating for their own care due to feeling that their practices were busy.

…but I just got the impression that my GPs, were just constantly overwhelmed and so I was the one that had to initiate lots of things I had to push for the blood tests and push for you do I need to chest X‐Ray and I was emailing my GP with you know the questionnaires, the list of things they should include on the referral. (P‐4)

GPs described the impact of time‐limited consultations and what can be covered:

Obviously we only have 10 minutes. There's (quite limits), a limit, to what we can do in 10 minutes. (HCP‐4, GP)

GPs commented that after multiple consultations they share the responsibility of care planning and timing of follow‐up appointments with patients. They noted that patients with more severe symptoms were more likely to return for multiple appointments.

I can't chase that, so you know it's up to them, you know the ones with more severe symptoms, I have two particular ones in my mind at the moment I've seen multiple times. Yeah, and they'll come back for review and will initiate some. antihistamines or … you know … famotidine and then they come back, and we'll review that and give them more information, bit by bit… (HCP‐4, GP)

#### 3.1.3 Specialist care

People with Long Covid described how they had needed to push for referral for further assessments or treatment from specialists, in a similar way as with their primary care physician.

Because it was me pushing I really feel as if me sending letters to people and pushing and chasing and making sure things happened I, the only reason I had the first MRI scan was because I kept chasing … after the Long Covid clinic letter I asked for that to be done, I had to ask again for it to be done, and then I was told that it would be done. (P‐6)

Throughout the interviews, people with Long Covid emphasised the huge efforts they needed to make to negotiate and access what they felt was appropriate care. These efforts incorporated active approaches, engaging with online communities, and pushing for their HCPs to facilitate access to various forms of care, including referrals, diagnostic investigations and treatment.

### 3.2 Patient–HCP interactions

Both patient and HCP participants reflected on how communication within the patient–HCP consultation could affect how people sought access for further care.

#### 3.2.1 Primary care

People with Long Covid described the importance of trust between them and HCPs, which develops through continuity of care, with their GP having prior knowledge of them. They described how continuity of care allowed HCPs to make more informed decisions about what appropriate care was for them and facilitated access to that care.

I think having the same doctor was a game changer for me. Just having that one person who was able to sort of remember what I had said before, was able to read his own notes. Adding that for me was so key. He's also really good. He seems to trust me when I bring him a problem … He referred me to the rapid diagnostics team where that happened very quickly… (P‐5)

GPs emphasised the importance of good communication with patients during consultations, which they felt facilitated shared decision‐making, framed as a shared responsibility, with GPs offering information about available treatments or interventions while respecting the patient's autonomy in determining the appropriate course of action for them:

…You know we'll sort of explore with them and give them an opportunity really to share their sort of concerns and where they're up to really and then depending on what happens then between the patient and us, we will then decide to look, you know what do you want to do next, what do you think you need? This is what we can offer. We think this will be good but it's up to you… (HCP‐3, GP)

People with Long Covid reported positive experiences when a GP had listened and supported a decision about treatment:

…was my current GP who has been open‐minded willing to take advice from me… and trying to make the rules work for the patients, as in giving me the off‐license medication, even though the budgets not there. (P‐7)

#### 3.2.2 Specialist care

People with Long Covid who had experienced a consultation with a specialist described the need to have their symptoms acknowledged and understood.

I went in they kind of checked everything I think it was the first time when a doctor sat with me for like 45 minutes and talked about my entire medical history every small, tiny little … And I think it's just it's it felt important to like to take a person and say ‘OK we're concerned, for your health. We're going to check you out we don't want you to be sick or die, we don't want to miss anything’ so that was that was really good. (P‐4)

This validation was also seen to be important by HCPs:

Listening to the patient is really important, that validation of their story and them being able to hear other people's stories and know that they're not the only person having the same, feeling the same way and have a same set of signs and symptoms, having access to somebody who can support them. And then having confidence so that they learn how to manage that, over time, so having that confidence and being able to manage their return to activity and knowing how to react to symptoms, so if they're feeling more unwell what to do about that, and how to know to progress so actually being able to have that self‐mastery of their condition going forward would be what I think we're really aiming for… (HCP‐6, specialist)

Setting expectations by specialists in terms of accessing care seemed to also be important.

We have some touch points so we manage, try and talk about expectations of how long we might have people in care for. So, we we'd talk about it being about three months, but most of our patients don't end up being with us just for three months, but for longer. But we're trying to manage the fact that people aren't going to stay with us forever and necessarily aren't going to stay with us until they're 100% better. (HCP‐6, specialist)

Both patients with Long Covid and HCPs emphasised the importance of building a trusting and collaborative relationship. Continuity of care, person‐centred care and trust were considered key factors leading to positive experiences of care.

### 3.3 Service resources and structural constraints

A recurring theme was the service resources and structural factors within the healthcare system that extend beyond patients and HCPs.

#### 3.3.1 Primary care

Even when a person recognised the possibility of Long Covid, various structural elements of the healthcare system were described as affecting the patients' ability to access primary care.

People with Long Covid reported barriers to accessing primary care, particularly long waiting times for a GP appointment:

I made a GP appointment again, and it was a three week wait for a GP appointment, because my GP is quite over‐subscribed. (P‐4)

Patients also described that the way appointments were held affected their abilities to attend them, especially brain fog which impacted patients' ability to access in‐person appointments. Telephone consultations were reported to be more acceptable to some people, because there was no requirement to travel.

They were initially remote. But I'm now traveling to appointments … before I moved. After the first lockdown I had to go to a GP appointment in person, I also to go for blood tests in person that I don't know how I done that, I honestly don't I drove. Shouldn't have driven because back then, I mean I was definitely dangerous to drive, but I did, and because of the brain fog. (P‐7)

Remote consultations, such as online communication and telephone consultations, were identified as possible facilitators to patients' healthcare journey.

I would check in with my GP once every two months to give him an update or something specific like when the digestive problems kicked in, call the GP practice, get a telephone appointment. He'd prescribe something like symptom control. I've had blood tests every six months, so you know, keep an eye out… (P‐5)

#### 3.3.2 Specialist care

There were potential problems with remote consultation noted by HCPs, in particular patients with poor digital literacy or lack of access to smartphone/laptop. A combination of in‐person and remote consultations was suggested as catering to different needs and to increase access to care.

Obviously knowing some people will be digitally challenged, don't have the technology uhm so we're hopefully mopping up everything. So, we can see people in the home or, we do that, or we might do a one to one remotely via video or telephone or we might bring them into an on‐site and see them maybe more you know in in a clinic type situation so, yeah so a very blended approach. (HCP‐6, specialist)

For specialist care, triaging referrals was reported to facilitate patients' access to appropriate level of care.

A single point of access now where all of those come through to us. We triage them on the basis of the self‐assessment questionnaire, which is done through an online portal and a phone conversation with our nurse navigator. Trying to refer people direct to post COVID rehab if that's safe but finding that more than two thirds of people need some sort of medically overseen assessment, because the patients that are getting referred are quite poorly. (HCP‐5, secondary care)

People with Long Covid, however, highlighted several structural constraints affecting their ability to access specialist appointments.

I had asked for referral by this stage to see a respiratory consultant, you know, and which I repeatedly chased and chased and chased and it took 14 months to get to see somebody you know… (P‐2)

Both patients and HCPs acknowledged the considerable waiting times for Long Covid services.

We still have 200 people waiting … so that's a capacity issue. (HCP‐2, secondary care)

There were facilitators that HCPs considered helpful during these waiting periods. Some services used self‐help psychological support services or self‐management services whilst waiting for specialist care.

Our waiting lists are down. So that's good we interact early on, with patients on the waiting list so we sign post them to self‐help information so patients can start looking at stuff and thinking about stuff they potential potentially can take up the [online therapy], or the [digital physical therapy] and so, although they are waiting a couple of months to see us, there are things that they can start doing if they want to so they're not just sitting there with nothing happening. (HCP‐3, GP)

It's just pointless, you know you can't leave somebody with Long Covid for six months without support, so we try to give them a triage call to you know put them in touch with some self‐management advice whilst they're waiting their appointment. (HCP‐5, secondary care)

None of the patients interviewed mentioned that they were offered psychological support during their wait for a specialist appointment. Some patients suggested that they would have appreciated communication from the specialist service whilst waiting.

Even if it was just a message saying your still on our list, don't worry we'll be in touch just so that you know. Because you know my referral went off in January, I didn't hear anything for months for the from the… (P‐8)

A lack of communication across services acted as a barrier for patients in obtaining referrals. This bureaucratic process was perceived to increase waiting times and led to a sense of frustration for people with Long Covid.

They couldn't even do the referral to a neurologist I have neurological symptoms, I clearly need to see a neurologist … but I wasn't really allowed to make an application sorry the GP wasn't allowed to do referral until we've been through Long Covid clinic. The Long Covid clinic couldn't even do the referral themselves all they did was write back to my GP to say make a referral to a neurologist, and so it was just a complete waste of many, many months. (P‐5)

HCPs however, suggested that if a patient's condition was more related to another specialty than Long Covid, they would coordinate with the relevant service and refer the patient onwards:

We're good at communicating … That's what we always do, and that's we have a patient, and if we need extra help we do talk to the people that we need help from so, it's not really anything new to us to do that, and so, and then, if we go in and somebody and think actually this is less Long covid and more their COPD, then we'd reach out to the COPD team and potentially hand that over and make that referral and ask them to be carried forward through the COPD team, so that that's how it's been managed at the moment. (HCP‐6, specialist)

These system challenges or facilitators affected patients' access to care and their overall healthcare experience.

**Figure 3:**
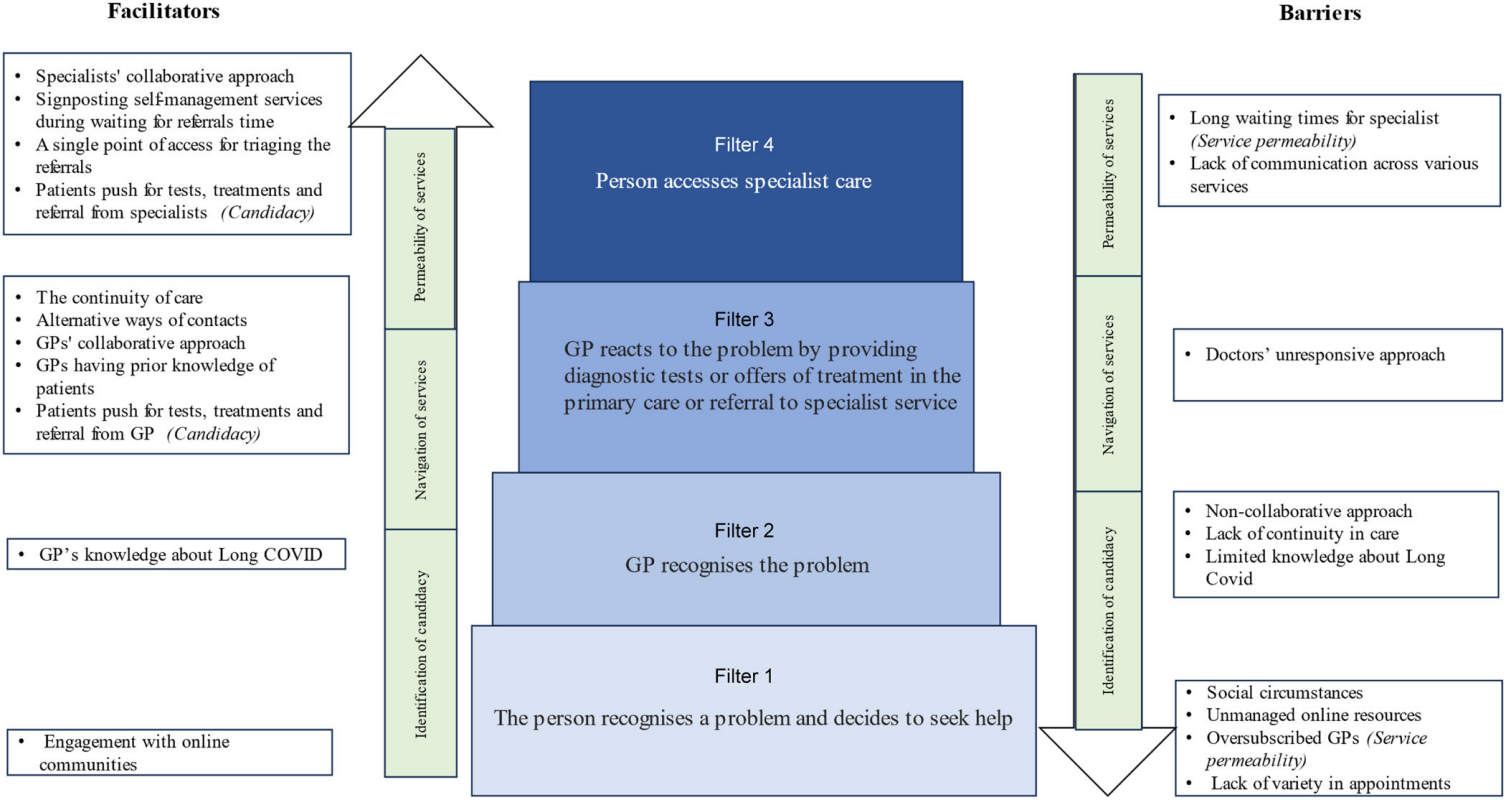
Integration of findings into the Pathways to Care Model and Candidacy Framework.

Overall, participants noted a range of systemic constraints impacting an individuals' access to, and experiences of, care. Social circumstances and long waiting times were identified as barriers, while blended appointment methods and booking systems were indicated as possible facilitators for accessing care. An overview of facilitators and barriers is provided in Figure 3.

In summary, both patients and HCPs reported similar facilitators and barriers in relation to service resources. They agreed that waiting times for specialists are excessively long, and alternative methods of contact were deemed helpful for both groups. However, while some HCPs mentioned signposting self‐management services during the waiting period for referrals, none of the patient participants mentioned being offered such services. Given the challenges around resources, people with Long Covid had to make significant efforts to negotiate pathways to access care. Both patients and HCPs emphasised the importance of building a trusting and collaborative relationship between HCPs and patients.

## 4 DISCUSSION

People with Long Covid reported the need to expend considerable efforts in pushing for referrals and treatment. Participants' identification of their own candidacy was pivotal to their access experience, and this was evident in our study by patients' engagement with online resources and communities. Our findings resonate with Ladds et al.^34^ who suggested that understanding and coping with a long‐term condition may become easier within peer support communities, which are often, though not exclusively, found online.^34^, ^35^ This was certainly the situation within our patient participants, where some found it to be their only source of support.

Appearance at healthcare services signifies the crucial step where individuals assert their claim to candidacy for care.^17^ Our study suggests that patients need to self‐advocate for themselves to navigate obstacles to secure access to care.^36^ For example, some people with Long Covid perceived that they were initially denied access but did not passively accept the situation; instead, they persisted and advocated for what they perceived as their ‘right’.^36^ Patient advocacy in Long Covid may play a crucial role in shaping the healthcare journey. This active involvement not only facilitates individual diagnosis and treatment, but also contributes to a broader impact by increasing awareness of Long Covid, given it is a relatively ‘new’ condition.

Limited understanding and knowledge within the healthcare settings of Long Covid and its complexities posed a barrier, but a collaborative, patient‐centred approach enhanced care. In line with previous qualitative research focusing on individuals experiencing Long Covid, our participants emphasised the importance of being believed to enhance the recognition of the condition,^37^ and a desire for the GP to believe in patient‐reported symptoms and express understanding and empathy.

Our findings support the pivotal role of continuity of care in general practice for people with long‐term conditions,^34^, ^38^, ^39^ extending this importance to people with Long Covid. Our participants' reported views and experiences suggest that a trusting and collaborative relationship between patients and HCPs plays a pivotal role in effectively managing Long Covid. This enhances the recognition of the patients' problem, informing the decision‐making process, and tailoring the care to meet their needs within a therapeutic relationship between patients and their GPs. Similar findings were provided in a study on primary care management of conditions including persistent pain.^39^ The situation can be exacerbated when care pathways are interrupted by discharges back to the GP for onward referrals, despite the contractual expectation that this should be done within the specialist provider service.^40^ Past experiences of care will influence future help‐seeking.^41^

Barriers to specialist care included long waiting times and communication gaps across services. The permeability of healthcare services, crucial for ease of access and utilization,^17^ becomes evident in instances where long waiting times act as barriers to timely care‐seeking potentially deterring individuals from accessing the necessary services promptly. Dixon‐Woods et al.^17^ noted that the negotiation stages highlighted the dynamic nature of the system, and more specifically, the constant negotiation between service users and HCPs.^17^ Navigation of healthcare services is not solely dependent on the willingness to seek care but also on an individual's knowledge of available services and their practical ability to access them. In this context, we found that people living with long Covid experienced a limited availability of appointments which served as a barrier to accessing primary care. Previous research has indicated that access to care is influenced by inequalities and structural constraints.^16^, ^42^

Overall, both the perspectives of patients and HCPs were usually aligned (e.g., long waiting lists as barriers or collaborative approaches as facilitators). Although peer support via online communities was reported to play a role by both HCPs and patients, practitioners highlighted that online misinformation may act as a barrier to swift support. This is due to HCPs needing to correct misinformation before informed decisions about care planning can be made. Strategies to mitigate online misinformation could involve improving communication between healthcare providers and patients, such as encouraging GPs to signpost patients to reliable and evidence‐based resources such as NHS and NICE websites.

Collaborative approaches and patients' efforts to push for treatments and tests were found to be relevant facilitators in both primary and secondary care settings (Figure 3). Although some specialist healthcare settings provided self‐management resources for people awaiting appointments, these were not widely available. Significant barriers in terms of waiting lists were more pronounced for secondary care compared to primary care. A lack of communication across different trusts, especially primary and secondary care, was identified as a barrier.

Overall, our themes—navigate, negotiate access, overcome structural/resource barriers—align well within the candidacy model,^17^ demonstrating multiple processes such as the identification of candidacy, negotiation, permeability and appearances at health services. These aspects seem to be especially important for the emerging new pathway model and are relevant to both primary and secondary care.

### 4.1 Strengths and limitations

Input from our patient advisory group strengthens this study. Interviewees who had responded to the nationally distributed survey for the Delphi study, which had 285 participants, indicating a representative sample, and had indicated that they were available for interview. No further selection had taken place. Nevertheless, in general people who are adept at self‐advocacy may be more likely to engage in research, suggesting that those who do not actively advocate for their care may be overlooked in terms of research participation. This could mean that people who had positive experiences were underrepresented, or individuals with more severe symptoms or limited digital literacy are unlikely to participate in research, leading to the exclusion of their perspectives from our findings. Therefore, it is possible that any tendencies at self‐selection may have evened out indicating limited selection bias. A strength of our research lies in the diverse locations of the interviewees across England, as they were not tied to specific healthcare settings. Also, there was good variety in terms of duration of Long Covid. People with Long Covid described a range of symptom duration spanning several months to more than a year, with symptoms either fluctuating or progressing. For instance, two patients had been experiencing symptoms since July 2020, while another patient had been living with fluctuating symptoms for 18 months and was not fully recovered. Additionally, three other patients reported symptom onset between September and November 2022, while the remaining patient had a shorter duration of around three to four months. This reflects the diversity in symptom duration properly.

However, the respondents available for interview were predominantly white British which suggests that the study findings may not be indicative of experiences from individuals with Long Covid from diverse backgrounds. This is important because the experiences of Long Covid care can be influenced by different cultural, racial or socioeconomic backgrounds. The historical struggles of minority communities with healthcare stigma, discrimination, and negative experiences, along with the fear of further stigmatisation, could contribute to the under‐representation of socioeconomically disadvantaged groups in Long Covid healthcare services.^43^ This may also reflect the challenges of health inequalities impacting on particular and marginalised groups in societies and communities.

The study sample was evenly distributed, with eight participants in each group; this seems a small sample, however, we ended our participant recruitment upon reaching thematic saturation from our interviews. Since the participants in this study were based in the UK, the healthcare experiences of people with Long Covid outside of the UK may be different to those described here, although it is likely core elements will be common across different healthcare systems and countries.

### 4.2 Implications

Overall, people with Long Covid described challenges in accessing consistent follow‐up care due to shared responsibility across various healthcare settings. Therefore, enhancing and sharing patient information among different institutions could facilitate improvements in the speed and relevance of care provision. Blended approaches to consultations (remote vs. face‐to‐face) might facilitate access, particularly the offer of remote consultations providing more convenient access for people with Long Covid struggling with fatigue and brain fog. Another approach, frequently found in integrated care models, involves appointing a single professional, often referred to as a care or case manager, to coordinate services and aid patients and their family members in navigating the healthcare system.^44^ The collaboration between primary and secondary care is considerably important, as fragmented healthcare services can contribute to patients' records being lost during the referral process, leading to delays in access to care and recovery.^45^

Integrated care pathways would also be effective in achieving continuity of care. Long Covid clinics, utilising a virtual multidisciplinary team, demonstrate enhanced collaboration, knowledge‐sharing, and integration of primary and specialist care, minimising the need for additional referrals to single‐specialty services and contributing to a more seamless and continuous patient care experience.^46^ Such integrated systems could overcome barriers related to the lack of communication across different care settings. To alleviate the waiting lists, initiatives within NHS England such as the promotion of digital outpatient transformation, encouraging personalised patient‐led follow‐up and implementing an advice and guidance facility, aim to provide swift responses, addressing GPs' inquiries within a short period.^47^

While NHS guidelines clearly state that specialists must refer onward if they are part of the same care pathway for a specific problem.^40^ The study found some instances where the guidelines may not have been followed. This indicates possible communication breakdowns, posing risks of delays and suboptimal patient outcomes. Adhering to established protocols is essential for ensuring timely and coordinated care.

Overall, considering the identified barriers in this study, policymakers could consider improving integrated healthcare systems to reduce waiting lists, enhance continuity of care and improve communication across different care settings. HCPs would benefit from collaborating with their patients, providing evidence‐based online resources and demonstrating reassurance and empathy. Further research could investigate access to care for people with Long Covid in diverse samples to address potential health inequalities.

## 5 CONCLUSION

Our findings reflect that, in the third year of the pandemic, people with Long Covid were still describing barriers to accessing care. There is *still* a need for people living with Long Covid to be believed by the practitioners they encounter. The collaborative, patient‐centred approaches, and continuity of care improved access to care. The perspectives of both patients and HCPs largely aligned; however, practitioners emphasised that online misinformation could hinder prompt support. This study highlights the need to focus on improving access and fostering collaboration and communication across different healthcare settings. Current findings can be further built upon by investigating ethnically diverse and socioeconomically disadvantaged populations and different healthcare settings.

## AUTHOR CONTRIBUTIONS

**Fidan Turk**: Writing—original draft; writing—review and editing; methodology; formal analysis; data curation. **Jennifer Sweetman**: Conceptualisation; methodology; writing—review and editing; writing—original draft; formal analysis; data curation. **Carolyn A. Chew‐Graham**: Writing—review and editing. **Mark Gabbay**: Conceptualisation; funding acquisition; writing—review and editing; methodology. **Jessie Shepherd**: Writing—review and editing; data curation. **Christina van der Feltz‐Cornelis**: Conceptualisation; funding acquisition; writing—review and editing; methodology; writing—original draft; data curation; supervision; project administration.

## CONFLICT OF INTEREST STATEMENT

Over the last 3 years, Christina van der Feltz‐Cornelis received funding for the European Platform to Promote Wellbeing and Health in the workplace (EMPOWER), a European project to reduce the impact of mental health problems at the workplace, from the European Union's Horizon 2020 research and innovation programme under grant agreement No. 848180. She received grants from The Netherlands Organization for Health Research and Development, grant number 537001002 and 5370010021, from NIHR, grant number 132852 and COV‐LT2‐0043, and from the BMA, for unrelated projects. She received royalties from several publishers for books on the topic of psychiatry. She received an honorarium from Janssen for speaking at a symposium and support for giving a lecture at the Lloyds Foundation annual conference 2019. Jennifer Sweetman, Fidan Turk and Jessie Shepherd received funding for EMPOWER from the European Union's Horizon 2020 research and innovation programme under grant agreement No. 848180. Christina van der Feltz‐Cornelis, Carolyn A. Chew‐Graham and Mark Gabbay declare receipt of NIHR funding for studies into Long Covid. Mark Gabbay has received NIHR and UKRI funding for research into applied health research.

## Supporting information

Supplementary information.

## Data Availability

The authors do not have permission to share data due to privacy and ethical restrictions.
